# Spatiotemporal and variability gait data in community-dwelling elderly women from Brazil

**DOI:** 10.1590/bjpt-rbf.2014.0157

**Published:** 2016-03-22

**Authors:** Renata N. Kirkwood, Henrique A. Gomes, Rosana F. Sampaio, Sheyla R. C. Furtado, Bruno S. Moreira

**Affiliations:** 1 Programa de Pós-graduação em Ciências da Reabilitação, Universidade Federal de Minas Gerais (UFMG), Belo Horizonte, MG, Brazil

**Keywords:** gait, elderly, principal component analysis, biplot, variability, physical therapy

## Abstract

**Background:**

Gait is an extremely complex motor task; therefore, gait data should encompass as many gait parameters as possible.

**Objective:**

To provide reference values for gait measurements obtained from a Brazilian group of community-dwelling elderly females between the ages of 65 and 89 years and to apply the PCA-biplot to yield insight into different walking strategies that might occur during the aging process.

**Method:**

305 elderly community-dwelling females living in Brazil were stratified into four age groups: 65-69 years (N=103); 70-74 years (N=95); 75-79 years (N=77); and ≥80 years (N=30). Age, height, and BMI were assessed to describe the characteristics of the groups. Gait spatiotemporal and variability data were obtained using the GAITRite® system. Principal component analysis, followed by MANOVA and the PCA-biplot approach were used to analyze the data.

**Results:**

95% CI showed that only three components – rhythm, variability, and support - together explained 74.2% of the total variance in gait that were different among the groups. The older groups (75-79 and ≥80 years) walked with lower than average velocity, cadence, and step length and were above average for the variables stance, step, swing, and double support time and the ≥80 year old group presented the highest gait variability compared to the other groups.

**Conclusion:**

Aging is associated with decreased gait velocity and cadence and increased stance, step time, and variability, but not associated with changes in base of support. In addition, the PCA-biplot indicates a decline towards decreased rhythm and increased variability with aging.

## Bullet points

- This study provides extended gait parameters for Brazilian elderly women.
- The components (rhythm, variability, and support) were different among age groups.
- Aging is associated with decreased gait rhythm and increased gait variability.
- Aging is not related to changes in base of support.

## Introduction

The normal aging process is associated with changes to the nervous, muscular, and skeletal systems that affect the ability of a person to walk efficiently^1^. These changes, specifically in the elderly population, have been associated with greater immobility, risk of falls, dementia, and mortality^2^ ^-^ ^5^. One approach to understanding gait dysfunction is to assess and compare the results with reference values^6^ ^-^ ^9^. Traditionally, gait velocity is the elected variable used to assess gait due to the link of lower gait velocity to adverse outcomes in the elderly^10^ ^,^ ^11^. However, gait is an extremely complex motor task that can be expressed from parameters other than velocity such as cadence, base of support, step length, swing, stance, and double support times. In addition, gait variability, defined as a fluctuation in gait parameters during a stride, is an important indicator of impaired mobility in the elderly^12^ ^,^ ^13^. Most studies focus on gait velocity, neglecting the other gait parameters^14^ ^,^ ^15^, however gait should also be recognized in other facets. To our knowledge, there is a lack of gait studies conducted in community-dwelling older adults living in Brazil.

The problem that arise from gathering many gait parameters is the data dimensionality, temporal dependence, and the high variability among these variables^16^. Therefore, the need for reduction and orthogonality of data is critical. One approach that has been successfully used in a large and correlated number of variables is Principal Component Analysis (PCA)^17^ ^,^ ^18^. PCA addresses these questions by reducing data dimensionality and maximally preserving data variance, in addition to generating a small set of orthogonal new variables^16^. This new set of variables or components represents a weighted linear combination of the original variables, which holds clinical features that can be interpreted and compared between groups. Moreover, the structure of the components can be interpreted using the PCA-biplot approach, which is a graphic display that gives insight into relationships, trends, and clusters between the variables and groups in the study^17^ ^,^ ^18^.

Therefore, the purpose of this article was twofold: 1) to provide extended values for gait measurements obtained from a Brazilian group of community-dwelling elderly females aged 65 to 89 and 2) to apply the PCA-biplot analysis to yield insight into different walking strategies that might occur during the aging process.

## Method

### Participants

A convenience sample of 305 elderly women was recruited from the community and from two primary care units in the city of Belo Horizonte, MG, Brazil. The inclusion criteria were: females; age ≥65 years; and ability to walk independently without assistive devices. The exclusion criteria were as follows: cognitive impairment detectable by the Mini-Mental State Examination (MMSE)^19^; visual impairments not corrected by lenses; musculoskeletal disorders (e.g. scoliosis); and motor sequel (e.g. stroke and Parkinson’s disease) that could affect gait. The sample was stratified into four age groups: 65-69 years (N=103); 70-74 years (N=95); 75-79 years (N=77); and ≥80 years (N=30). Anthropometric data included height (cm), mass (kg), and body mass index (kg/m^2^). All of the participants signed the informed consent form approved by the research ethics committee of the Municipal Health Department of Belo Horizonte, MG, Brazil (protocol number: 0081.1.410.000-09A).

### Gait assessment

Spatiotemporal gait parameters and variability measures were collected at preferred walking speed using a 5.74 m computerized carpet (GAITRite®, CIR Systems Inc., Havertown, PA, USA) placed on a well-illuminated hallway and free of noise and visual distractions. Participants wore their own closed, low-heel footwear and performed six walks (on average 4 to 5 steps long) beginning and stopping 2m before and after the carpet to allow for the acceleration and deceleration phases. Data was combined into a single test, sampled at 120 Hz, and processed using the system software including: velocity (cm/s), cadence (steps/min), step length (cm), base of support (cm), step time (s), swing time (s), stance time (s), and double support time (s) as defined by the GAITRite^®^ manual. Coefficient of variation (CV = [standard deviation/mean] × 100) was used as a measure of gait variability for the following parameters: velocity (%CV), step length (%CV), base of support (%CV), step time (%CV), swing time (%CV), stance time (%CV), and double support time (%CV).

### Data analysis

Anthropometric data were presented descriptively. PCA summarized the variation in a multi-correlated data (15 gait variables) to a set of uncorrelated components. The extracted uncorrelated principal components (PC) are equal to the number of variables and are estimated from eigenvectors of the covariance matrix of the original variables^20^. The PCs are the linear combination of the 15 standardized variables and are presented in decreased order of importance^18^ ^,^ ^21^. The lack of correlation between the PCs means that each PC measures a different feature of variance within the original data^18^. The relative weighting of the original variables in each component contributes to the clinical interpretation of each PC, and the sign indicates the nature of the correlation between the variable and the PC^18^ ^,^ ^22^ ^,^ ^23^. Variables with a contribution ≥0.30 were considered for the interpretation.

The analysis also gives a set of scores that represents the distance each individual is from the mean score of each component^18^ ^,^ ^24^ ^,^ ^25^. The resultant PC scores were standardized to *z* scores (mean of zero and standard deviation of 1) and 95% confidence intervals (CI) were generated to determine which PCs were different between the groups.

Next, MANOVA was conducted with the primary contributing variables (weight coefficient ≥0.30) of the significant components – indicated by the 95% CI – to determine which variables were different between the groups. A post hoc Bonferroni correction was conducted for multiple comparisons.

A PCA-biplot was built to interpret the relationship between the PCs, the scores, and the variables^18^. The PCA-biplot has its axes represented by the PC loadings, the average of the PC scores of each groups represented by symbols, and the variables represented by vectors scaled to have a unit length in the original dimensional space. Interpretation involves understanding how the groups are represented in this 2-PC model and what each PC means in terms of the original variables. The length of the variables’ vectors indicate its relative variance and the direction with respect to the axes indicates the PC to which each variable is most strongly related. When the projection of the observation (perpendicular line from the observation to the variable vector) falls in the direction of the variable, it means that the group has a higher than average value for that specific variable; when it is in the opposite direction of the vector, the value is lower than average^17^. All tests were analysed using SPSS 22.0 (SPSS Inc., Chicago, IL, USA) and MatLab (R2011a) with a 0.05 significance level.

## Results

The characteristics of the study groups are summarized in Table 1 and the reference values of the gait variables for the different age groups are shown in Table 2. PCA resulted in four components, with eigenvalues greater than 1, that explained 81.7% of the total variance (Table 3). PC1 explained 43.2% and was heavily loaded with the variables velocity and cadence going in a positive direction and stance and step time going in opposite directions. Therefore, this component was labelled ‘rhythm’ because changes in either pair of variables would affect the repeated pattern of the gait cycle. PC2 explained 19.1% and was loaded only with gait variability data, all going in the same positive direction (velocity, step time, stance time, and double support time); thus, the component was named ‘variability’. PC3, with 11.9% of variance explained, was labelled ‘support’ due to the weight contribution of the variables base of support and base of support variability going in opposite directions. Therefore, an increase in base of support length decreases base of support variability, and the opposite is true. The last component PC4 explained 7.5% and was loaded with the variables double support time and variability and swing time; therefore this component was named phases. The 95% CI of the PCs showed that only three components – rhythm, variability, and support –were difference among the groups and together explained 74.2% of the total variance. Therefore, the remainder of the analysis was conducted only on the significant components.

**Table 1:** Anthropometric characteristics of the groups (N=305).

Anthropometrics	Group 65-69 years N=103	Group 70-74 years N=95	Group 75-79 years N=77	Group ≥80 years N=30
Age (years)	67.3±1.3	72.0±1.4	76.7±1.4	82.7±2.5
Height (cm)	154.4±5.9	153.7±6.1	152.4±7.6	151.3±6.6
BMI (kg/m^2^)	27.7±4.4	27.5±4.2	27.5±4.8	25.7±4.0

BMI: body mass index.

**Table 2:** Reference values by group (Mean±SD and Range) of the gait parameters investigated in the study.

Gait Parameter	Group 65-69 years N=103	Group 70-74 years N=95	Group 75-79 years N=77	Group ≥80 years N=30
Velocity (cm/s) Range	128.5±18.4 85.4-168.9	121.4±18.2 64.5-171.2	115.1±18.5 73.6-158.1	105.4±23.4 65.7-155.9
Cadence (steps/min) Range	119.6±9.1 98.0-142.6	118.4±10.0 92.1-148.0	115.5±10.0 94.0-137.9	113.0±11.8 87.6-134.2
Step Length (cm) Range	63.1±6.2 48.5-77.7	61.4±6.3 42.0-72.2	59.6±6.7 0.44-73.2	55.5±7.8 39.0-71.9
Base of Support (cm) Range	7.5±2.3 2.3-14.9	7.8±2.4 2.0-13.0	7.6±2.8 2.4-17.5	8.1±2.5 2.0-13.0
Step Time (s) Range	0.50±0.04 0.42-0.61	0.51±0.04 0.41-0.65	0.52±0.05 0.44-0.64	0.54±0.06 0.45-0.69
Swing Time (s) Range	0.42±0.04 0.35-0.51	0.41±0.03 0.52-0.52	0.40±0.05 0.34-0.51	0.42±0.04 0.36-0.58
Stance Time (s) Range	0.60±0.05 0.48-0.74	0.61±0.06 0.49-0.85	0.63±0.06 0.53-0.81	0.65±0.08 0.52-0.80
Double of Support Time (s) Range	0.20±0.04 0.07-0.31	0.21±0.04 0.14-0.40	0.22±0.05 0.16-0.38	0.22±0.05 0.12-0.35
Velocity (%CV) Mean SD (cm/s)	3.9±1.3 4.9	4.4±1.8 5.3	4.7±1.8 5.3	6.0±3.7 6.0
Step Length (%CV) Mean SD (cm)	3.1±1.4 2.0	3.3±1.2 2.0	4.0±1.7 2.3	4.6±2.2 2.5
Base of Support (%CV) Mean SD (cm)	31.3±16.3 2.1	30.6±18.7 2.1	37.3±28.5 2.4	38.8±35.1 2.5
Step Time (%CV) Mean SD (s)	2.9±0.9 0.01	3.2±1.1 0.02	3.2±1.1 0.02	4.5±2.4 0.02
Swing Time (%CV) Mean SD (s)	3.4±1.1 0.01	3.7±1.3 0.02	3.8±1.1 0.02	5.0±2.0 0.02
Stance Time (%CV) Mean SD (s)	3.1±1.5 0.02	3.3±1.3 0.02	3.5±1.6 0.02	4.6±2.4 0.03
Double Support Time (%CV) Mean SD (s)	8.3±4.2 0.02	8.2±3.0 0.02	8.3±2.3 0.02	11.4±4.3 0.03

SD: standard deviation; CV: coefficient of variation; s: seconds; cm: centimeters.

**Table 3:** Loading vectors showing the variables with highest contribution (≥0.30) to each principal component and the percentage of total variation.

**Variables with contribution ≥0.30**	Loading Vectors			
	PC1^*^	PC2^*^	PC3^*^	PC4
**Rhythm**				
Velocity (cm/s)	0.33			
Cadence (steps/min)	0.32			
Stance Time (s)	–0.33			
Step Time (s)	–0.33			

**Variability**				
Stance Time (%CV)		0.38		
Double Support Time (%CV)		0.35		
Velocity (%CV)		0.31		
Step Time (%CV)		0.30		

**Support**				
Base of Support (%CV)			0.61	
Base of Support (cm)			–0.60	

**Phase**				
Double Support Time (%CV)				0.52
Swing Time (s)				0.42
Double Support Time (s)				–0.51
**Cumulative percentage of total variation (%)**	43.2	62.3	74.2	81.7

* 95% CI of the PC scores statistically significant between groups.

**Figure 1:**
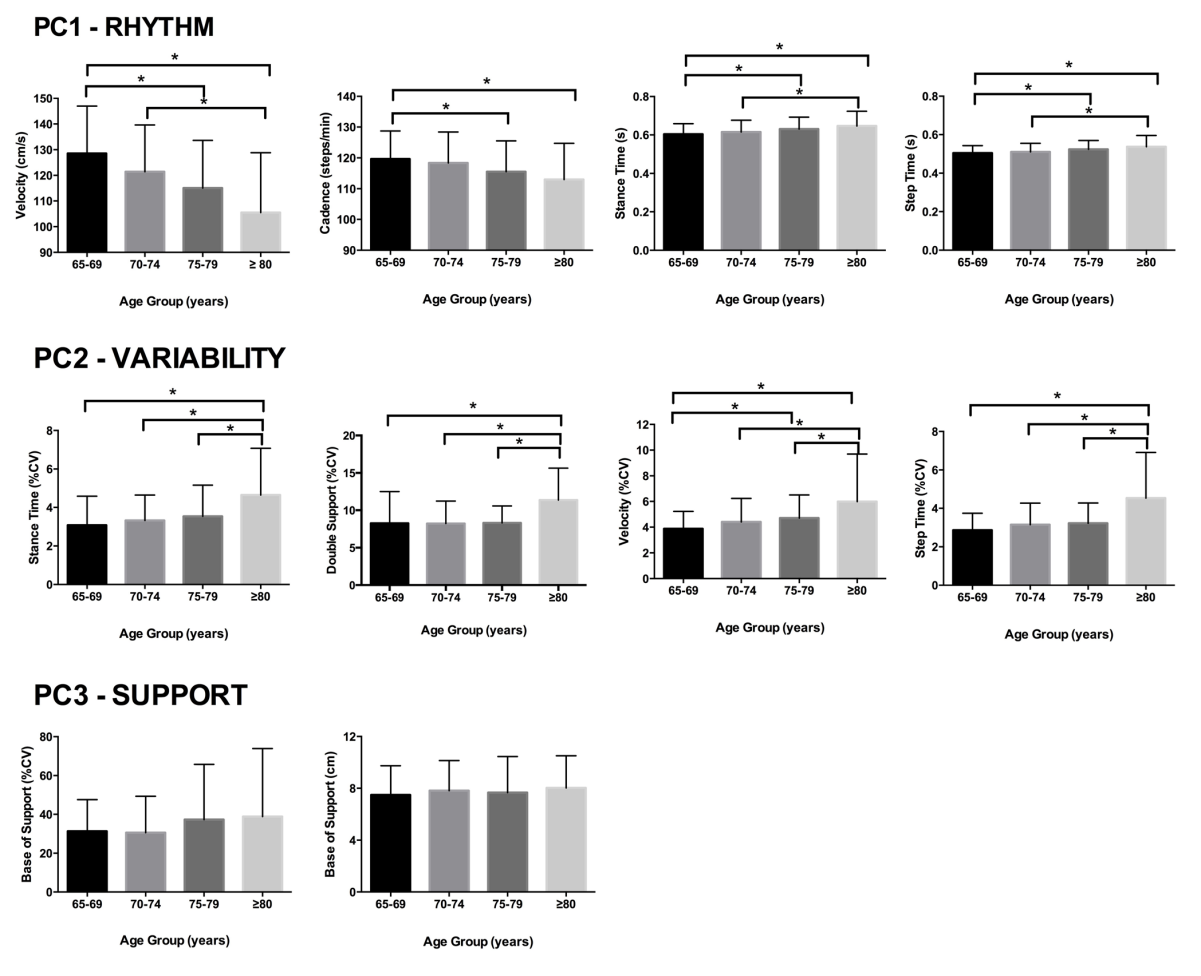
Comparison between groups (N=305) of the variables with greater contribution to the significant principal components. *: significant difference between groups.

Figure 1 shows the post hoc Bonferroni for multiple comparisons for the primary outcomes. There was a significant effect on groups for the primary outcomes of the components rhythm, variability, and support (F(10, 292) = 3.47, *p*<0.05). For the component rhythm (PC1), gait velocity was significantly greater in the 65-69 and 70-74 year old groups compared to the ≥80 year old group and in the 65-69 year old group compared to the 75-79 year old group. Cadence was significantly greater in the 65-69 year old group compared to the 75-79 and ≥80 year old groups. Stance time was significantly smaller in the 65-69 and 70-74 year old groups compared to the ≥80 year old group and in the 65-69 year old group compared to the 75-79 year old group. Similarly, step time was significantly smaller in the 65-69 and 70-74 year old groups compared to the ≥80 year old group and in the 65-69 year old group compared to the 75-79 year old group.

Post hoc comparisons for the component variability (PC2) showed that stance time, double support time, and step time variability were significantly smaller in the 65-69, 70-74, and 75-79 year old groups compared to the ≥80 year old group. The variability of gait velocity was significantly smaller in the 65-69, 70-74, and 75-79 year old groups compared to the ≥80 year old group and in the 65-69 year old group compared to the 75-79 year old group. The variables of the component support (PC3), i.e. base of support and base of support variability, showed no significant difference between groups.

**Figure 2:**
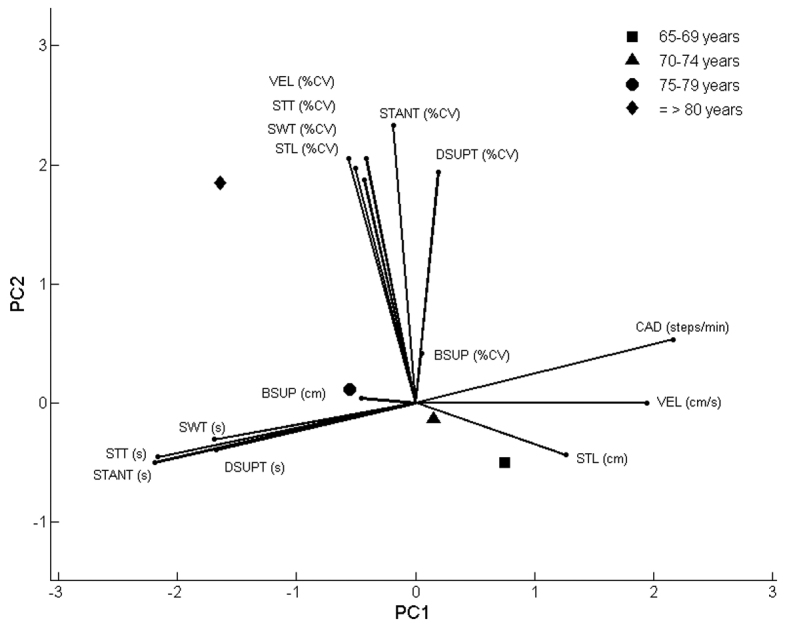
PCA-biplot and the average score of the groups (N=305). PC1=principal component 1; PC2=principal component 2; VEL=velocity; STT=step time; SWT=swing time; STL=step length; STANT=stance time; DSUPT=double support time; CAD=cadence; BSUP=base of support; %CV= coefficient of variation.

Figure 2 shows the PCA-biplot that displays PC1 on the x-axis and PC2 on the y-axis, with the average of the PC scores for each groups represented by symbols and the original variables shown by the vectors. The PCA-biplot clearly shows that gait spatiotemporal parameters were strongly related to PC1 and gait variability was strongly related to PC2. Base of support and base of support variability are poorly represented in PC1 and PC2, as expected. The proximity of the gait variables related to time showed a strong correlation among these variables as well as the proximity of the variability data. The longest variance is attributed to the variables cadence, step time, stance time, and stance time variability. The projection of the 65-69 year old group onto the variables shows that, on average, this group walked faster in relation to the other groups with higher cadence, velocity, and step length and reduced stance, swing, step, and double support time. The 70-74 year old group walked faster than the two other older groups but slower than the 65-69 year old group. The projection of the 75-79 year old group onto the variables shows that this age group walked with lower-than-average velocity, cadence, and step length and above-average stance, step, swing, and double support times. The projection of the oldest group (≥80 years) onto the variables clearly shows that this group had the lowest gait velocity with the highest time spent in step, stance, swing and double support times.

In relation to the variability, it is also clear that the ≥80 year old group presented the highest gait variability compared to the other groups. The PCA-biplot also shows that, with age, there is a tendency towards decreased gait velocity, increased time spent on stance and double support, and higher gait variability.

## Discussion

This study confirms the deleterious effect of age on gait parameters in a group of community-dwelling elderly women living in Brazil. The PC analysis was able to identify three components – rhythm, variability, and support – that clearly show that, in these groups of elderly women, aging is associated with decreased gait velocity and cadence and increased stance, step time, and variability, but not associated with changes in base of support. In addition, the PCA-biplot indicates a decline towards decreased rhythm and increased variability with aging.

Of the 15 gait variables entered in the analysis, four variables – velocity, cadence, step time, and stance time – contributed heavily to PC1. This means that reduction was obtained and the component could be clinically interpreted. Aging was associated with decreased rhythm characterized by lower velocity and cadence and increased step and stance time. The average velocity of the younger group (65-69) was 128.5 cm, which was 5.6% faster (difference of 7.1 cm/s) than the 70-74 year old group, 10.4% faster (difference of 13.4 cm/s) than the 75-79 year old group, and 18% faster (difference of 23.1 cm/s) than the eldest group (≥80 years). However, the differences between the 65-69 and the 70-74 year old groups (difference of 7.1 cm/s) and between the latter and the 75-79 year old group (difference of 6.3 cm/s) did not reach statistical significance. Significant changes for gait velocity reported by Brach et al.^26^, in a sample of community-dwelling older adults, were 4.15 cm/s for a small change and 10.38 cm/s for a substantial change. Therefore, the gait differences found in our study are substantial and deserve attention given that the adverse outcomes of decreased gait velocity with falls^4^ ^,^ ^27^ and fear of falling^28^ in the elderly^10^ have been well established. In addition, our data will help clinicians detect substantial gait velocity changes in older adults; thus, interventions aimed to improve gait velocity could be evaluated properly.

The older age groups (75-79 and ≥80 years) decreased rhythm by increasing time spent in stance and in step that culminated in decreased cadence and velocity. The greatest difference was 8.3% (difference of 0.05 s) in stance time between the 65-69 and ≥80 year old groups, and the smallest, but also significant, was 4% (difference of 0.02 s) in step time between the 65-69 and the 75-79 year old groups. A greatest difference in cadence was 5.6% (difference of 6.6 steps/min), observed between the 65-64 and ≥80 year old groups. Interestingly, stance and step times and cadence were very similar between the groups 70-74 and 75-79 years, and the PCA-biplot also shows that these groups are closer to the average of the variables’ values – with further proximity of the 70-74 year old group – but on opposite sides of the biplot. We could speculate that, in the absence of any disease impairment, the greatest changes in gait are expected to occur between the ages of 70 and 74 or that would be the transitional age for gait abnormalities due to aging. By transitional, we speculate a decreased gait velocity and increased variability. Decreased gait rhythm has been associated with dementia and a decline in cognition in a cohort study conducted with individuals older than 70 years of age^29^. In our study, the MMSE was the only test used to screen the subjects for cognitive impairments and no criteria was used to detect signs of early dementia. Therefore, it is possible that our participants could have mild cognitive impairment. However, all of them were capable of understanding the instructions of the study. Nevertheless, a decrease in rhythm was observed with aging with significant differences between the groups, highlighting the importance of rhythm in the process of evaluating elderly individuals.

The average gait velocity observed in our study was higher than the studies that reported gait parameters in Brazilian community-dwelling elderly females. Ruggero et al.^14^ reported 111 cm/s (SD: 27 cm/s) in a group of elderly women aged 65 to 92, and Novaes et al.^15^ reported 107 cm/s (SD: 17 cm/s) and 102 cm/s (SD: 10 cm/s) in a similar group between the ages of 60-69 and ≥70 years of age, respectively. The differences may be related to the measurement system used and the range of ages investigated. In our study, a computerized carpet with accepted validity^30^ and reliability^31^ was used to assess gait parameters, and in the previously mentioned studies, gait velocity was measured using a stopwatch, which could be a potential source of human error due to the uncertainty of determining the beginning and end of the cycle. In addition, our groups were stratified into 5-year ranges, with the exception of the ≥80 year old group. Therefore, our study provides extensive gait data over a wider range of ages – from 65 to 89 – and used a reliable measurement system.

Gait variability is considered a useful marker in predicting falls in elderly individuals^4^ ^,^ ^13^ ^,^ ^32^. The finding of a component loaded with gait variability parameters was expected and similar results have been described in the literature^6^ ^,^ ^7^ ^,^ ^29^. This means that after rhythm has been accounted for, the main source of variation in the data came from stance time, double support time, velocity, and step time variability, composing the component variability. The eldest group (≥80 years) presented the highest stance time, double support time, velocity, and step time variability compared to the other age groups. Callisaya et al.^13^ found a strong association between step time variability and older age in women (71.6±7.1 years), supporting our findings. The authors also pointed out that reduced gait velocity might be responsible for the increase in variability in gait parameters observed in elderly individuals^13^. This affirmation is also supported by our findings. The PCA-biplot clearly shows that the oldest groups (75-79 and ≥80 years) located on the extension of the velocity vector (negative side - lower-than-average velocity) are also the ones closer to and on the direction of the variability vectors (positive side - higher variability).

The component support was heavily loaded with the variables base of support and base of support variability, going in opposite directions. Thus, as base of support decreases, variability increases and the opposite is true. However, these variables failed to reach significance when compared between groups. The major difference in base of support was 8% (difference of 0.6 cm) found between the 65-69 and ≥80 year groups. In our experience, base of support is not a marker for differentiating older individuals^17^ ^,^ ^28^. Conversely, Brach et al.^33^ found that excessive step width variability, either too much or too little, in a non-challenging situation and at near normal gait velocity could be an early indication of fall risk in highly mobile individuals. Therefore, further studies are necessary to explain the effect of aging on base of support and base of support variability.

In the present study, double support time, double support time variability, and swing time comprised the component ‘phases’, but showed no difference between groups. Most of the studies^7^ ^,^ ^29^ that identified a significant phase component were based on factor analysis. One of the advantages of principal component analysis (PCA) over factor analysis is the amount of variance of the observed variables that is present in the components. While principal components explore a representation of the variance among the data, factor analysis seeks an efficient representation of the covariance among variables^16^. In factor analysis, the variance of a single variable is separated into common and error variances. The common variance is shared by other variables, but the error is unique to the particular variable. In PCA, the observed variables are summarized and the total variance makes no distinction between common and error variances^34^. Therefore, PCA accounts for the maximum variance present in the original variables with a minimum number of PCs. The principal components that account for large amounts of variance represent the majority of the variance of the data, and the principal components that account for a small amount of variance indicate random noise^21^. Since the experiments conducted were relatively error free, the error variance represents a small portion of the total variance; therefore, we believe that PCA is more appropriate for this type of study. Since most of the variance was accounted for and reduction was achieved within the first three components (74.2%), the contribution of the fourth component (phases) was minimal and probably not relevant.

One of the limitations of the study was the inclusion of a greater age range of elderly individuals in the ≥80 year group, from 80 to 89 years of age. The decision was taken to avoid groups with a small sample size, but we understand that we missed the opportunity to understand even more the effects of aging on gait parameters. Another factor to be considered is that physical activity level was neglected in the present study. We also understand that, for a study on elderly women, the screening criteria should be expanded to avoid the effects of sensory, cognitive, and mental impairments on gait.

## Conclusion

In conclusion, the present study provides extensive gait data on Brazilian community-dwelling elderly women between the ages of 65 and 89. Through a robust statistical analysis, the effects of aging on gait rhythm and variability were described, and the information will contribute to the assessment and treatment of elderly individuals.
